# Supplementary material for: The introduction of the Barbier reaction into polymer chemistry
Source: Nat Commun. 2017 Oct 31;8:1210. doi: 10.1038/s41467-017-01472-w (PMC5662735; doi:10.1038/s41467-017-01472-w)
Supplement: Supplementary file 1 — Supplementary Information [file 41467_2017_1472_MOESM1_ESM.pdf]

Supplementary Methods

Materials. THF was distilled from Na/benzophenone prior to use. All other solvents and chemicals used throughout the experiment were analytical-grade and were used without further purification.

Characterizations.

Nuclear Magnetic Resonance (NMR) Spectra. The ^{1}H and ^{13}C NMR spectra were recorded in $CDCl_3$ solution on Bruker AscendTM 400 NMR spectrometer (400 MHz for ^{1}H, and 100 MHz for ^{13}C) using internal tetramethylsilane as an reference.

Fourier transform infrared (FT-IR) spectroscopy. FT-IR Spectra using KBr pellets were recorded on a TENSOR II FTIR Spectrometer (Bruker, Germany). The spectra were recorded from an accumulation of 16 scans in the range of 4000 - 400 cm^{-1} and were collected at room temperature. The OPUS v7.5 software was used to auto-correct the spectral base line and calculate the second derivative spectra.

X-ray photoelectron spectroscopy (XPS). XPS studies were carried out on an ESCALAB 250 XI XPS instrument (Thermo Scientific, USA) in an ultra-high vacuum system using monochromated Al K_α (150 W, 20 eV pass energy) radiation. The spectra were recorded at 100 eV pass energy with the step size of 1.00 eV for the survey scan. The peaks were fitted and analyzed using ThermoScientific Avantage 4.75 software.

Gel permeation chromatography (GPC). The molecular weight and molecular weight distribution were determined on a Viscotek TDA 302 triple detector array equipped with one TSK-Gel GMH_{HR}-N column at 30 ℃, and THF was used as eluent

at a flow rate of 1.0 mL/min. Monodispersed polystyrene standards were used in the calibration of molecular weight and molecular weight distribution.

Static light scattering (SLS). SLS characterizations were conducted with a commercial light scattering spectromter (model：ALV / CGS-3 Compact Goniometer) equipped with an ALV/LSE-5004 light scattering electronics, a multi-t digital time correlator, a JDS-Uniphase 22 mW He－Ne laser (at λ = 632.8 nm) at 25 ^{o}C and the angles (θ) ranging from 15° to 150°. Polymer solutions with the concentration of 6.34 mg/mL was prepared in toluene 24 h ahead and filtered through a 0.22 mm PES membrane prior to characterization. The dn/dc value used was 0.0935 mL/g, which was measured through Orange dn/dc refractometer.

UV-vis absorption spectra of solution were recorded in THF with sample concentration of 20 μg/mL and sample solution transmittance at 500 nm wavelength were acquired in THF/Water mixtures with different water fractions (vol%) on a Shimadzu UV-2450 UV-Vis spectrophotometer at room temperature. The UV-vis absorption spectra of solid samples were recorded on Hitachi U-3010 spectrophotometer with an integrating sphere facility, using barium sulfate as reference.

The fluorescence spectra of all samples were acquired in a quartz cuvette with a path length of 1 cm using a Hitachi F2500 fluorescence spectrofluorometer. The absolute fluorescence quantum yield was measured with an integrating sphere on an Edinburgh Instruments FLS980 three-monochromator spectrophotometer.

Thermo-responsive measurements of the polymer solution (2 mg/mL) in DMSO, DMF and THF with different amounts of water (vol%) based on turbidity were performed using an oil bath with a digital thermo-controller, where the temperature with an obvious turbidity change in the heating process was used to determine the UCST. The fluorescence emission spectra and digital photos of the polymer solution (2 mg/mL) in DMF with 45% of water (vol%) at different temperatures were acquired respectively on Hitachi F2500 fluorescence spectrofluorometer, under sunlight and irradiation with UV lamp 365 nm.

Aggregation-induced emission (AIE) measurements of the polymer in solvent/nonsolvent mixtures were tested. In this study THF was selected as good solvent for polymers and water was selected as aggregation-inducing nonsolvent. The fluorescence spectra and transmittance at 500 nm wavelength of polymers in THF/Water mixtures with different water content were recorded respectively on a Hitachi F2500 fluorescence spectrofluorometer and on a Shimadzu UV-2450 UV-Vis spectrophotometer. The absolute fluorescence quantum yield of polymers in THF/Water mixtures with different water content was measured with an integrating sphere on an Edinburgh Instruments FLS980 three-monochromator spectrophotometer. AIE measurements of the polymer on thin layer chromatography plate was tested by tracing the digital photos of one drop of PTPM-1 solution (10 mg/mL in THF) on thin layer chromatography plate with different evaporation timescale at room temperature (under irradiation with UV lamp @ 365 nm).

The detection of 2,4,6-trinitrotoluene (TNT) was performed by fluorescence quenching of aggregated polymer in the THF/Water mixtures. The fluorescence spectra (excitation @ 346 nm) of polymer solution (0.02 mg/mL in water/THF mixtures with 90% water fractions (vol%)) upon addition of different amounts of TNT were tested. Thus the intensities and quenching ratio (the fluorescence quenching efficiency = (1- I/I_0) ×100%, I and I_0 denote the fluorescence intensity of polymer with and without TNT, respectively) of polymer solution upon addition of different amounts of TNT were acquired. Further, Corresponding Stern–Volmer plots of TNT was acquired, which can be used to detect the content of TNT.

Luminescent polymer film formation was carried out through evaporation of a THF solution of polymer on the quartz plate**.** RB/polymer film was prepared through evaporation of a THF solution of polymer and RB (1 %, wt %) on the quartz plate. RB film was acquired by evaporation of a THF solution of RB on the quartz plate.

The preparation of luminescent PTPM-1 textile through electrospinning. The electrospinning precursor solutions were prepared by dissolving the PTPM-1(0.851 g) in 5 ml DMF, which was stirred for 12 h at room temperature. After that, the solution was loaded into 5 ml plastic syringes with a 20-gauge needle. The electrospinning apparatus used in this work was the ET-2535H spinning equipment (Beijing Ucalery Technology Development Co., Ltd., China). A high voltage of 20 kV was applied onto the needle. The distance between the needle and collector was 18 cm, and the solution feed rate was set at 1 mL/h. The luminescent textile were deposited on the tinfoil and then dried overnight under vacuum to remove the residual solvent.

Synthesis

Synthesis of polytriphenylmethanol (PTPM-1). To one flame-dried 2-neck round bottom flask containing 0.288 g (12.0 mmol) of freshly peeled Mg scraps, was added 20 mL of dry THF. Then 2.611 g (10.0 mmol) 4-bromobenzophenone dissolved in 10 mL THF was added to the flask at room temperature through a syringe. After being stirred for 5 min, 0.1 mL of 1, 2-dibromoethane was added to the flask as activator. After the reaction was refluxed for 24 h, the solution was cooled to room temperature, followed by quenching and hydrolysis with 20 mL saturated aqueous ammonium chloride. After filtration and workup with dichloromethane/water, the organic solution was dried with anhydrous $MgSO_4$ and concentrated under reduced pressure. After the product was purified by precipitation into excessive petroleum ether, filtered and dried under vacuum, 1.579 g PTPM-1 was obtained as a yellow powder with a yield of 86.4 %. ^{1}H NMR (400 MHz, $CDCl_3$): δ=7.89-7.21(broad, -C6H5, 5H), 7.21-6.76 (broad, -C6H4-, 4H), 3.19-2.92 (broad, -OH, 1H). ^{13}C NMR (100 MHz, $CDCl_3$): δ=196.31, 146.64, 143.85, 128.65, 127.86, 127.33, 126.55, 82.86.

Synthesis of polytriphenylmethanol (PTPM-1'). To one flame-dried 2-neck round bottom flask containing 0.288 g (12.0 mmol) of freshly peeled Mg scraps, was added 20 mL of dry THF. Then 2.167 g (10.0 mmol) 4-chlorobenzophenone dissolved in 10 mL THF was added to the flask at room temperature through a syringe. After stirring for 5 min, 0.1 mL of 1, 2-dibromoethane was added to the flask as activator. After the reaction was refluxed for 24 h, the solution was cooled to room temperature, followed by quenching and hydrolysis with 20 mL saturated aqueous ammonium chloride.

After filtration and workup with dichloromethane/water, the organic solution was dried with anhydrous $MgSO_4$ and concentrated under reduced pressure. After the product was purified by precipitation into excessive petroleum ether, filtered and dried under vacuum, 1.527 g PTPM-1' was obtained as a yellow powder with a yield of 83.6 %. ^{1}H NMR (400 MHz, $CDCl_3$): δ=7.67-7.22 (broad, -C6H5, 5H), 7.22-6.77 (broad, -C6H4-, 4H) , 3.34-3.05 (broad, -OH, 1H). ^{13}C NMR (100 MHz, $CDCl_3$): δ=196.96, 145.82, 144.08, 133.18, 131.89, 129.96, 128.46, 83.52.

Synthesis of polytriphenylmethanol (PTPM-2). To one flame-dried 2-neck round bottom flask containing 0.288 g (12.0 mmol) of freshly peeled Mg scraps, was added 20 mL of dry THF. Then 2.611 g (10.0 mmol) 3-bromobenzophenone dissolved in 10 mL THF was added to the flask at room temperature through a syringe. After stirring for 5 min, 0.1 mL of 1, 2-dibromoethane was added to the flask as activator. After the reaction was refluxed for 24 h, the solution was cooled to room temperature, followed by quenching and hydrolysis with 20 mL saturated aqueous ammonium chloride. After filtration and workup with dichloromethane/water, the organic solution was dried with anhydrous $MgSO_4$ and concentrated under reduced pressure. After the product was purified by precipitation into excessive petroleum ether, filtered and dried under vacuum, 1.340 g PTPM-2 was obtained as a white powder with a yield of 76.6 %. ^{1}H NMR (400 MHz, $CDCl_3$): δ=7.89-7.18 (broad, -C6H5, 5H), 7.18-6.74 (broad, -C6H4-, 4H) , 3.77-3.32 (broad, -OH, 1H). ^{13}C NMR (100 MHz, $CDCl_3$): δ=187.14, 141.41, 140.81, 130.08, 129.14, 128.63, 128.29, 127.28, 126.94, 87.78.

Synthesis of polytriphenylmethanol (PDPM). To one flame-dried 2-neck round bottom flask containing 0.288 g (12.0 mmol) of freshly peeled Mg scraps, was added 20 mL of dry THF. Then 1.990 g (10.0 mmol) 4-bromoacetophenone dissolved in 10 mL THF was added to the flask at room temperature through a syringe. After stirring for 5 min, 0.1 mL of 1, 2-dibromoethane was added to the flask as activator. After the reaction was refluxed for 24 h, the solution was cooled to room temperature, followed by quenching and hydrolysis with 20 mL saturated aqueous ammonium chloride. After filtration and workup with dichloromethane/water, the organic solution was dried with anhydrous $MgSO_4$ and concentrated under reduced pressure. After the product was purified by precipitation into excessive petroleum ether, filtered and dried under vacuum, 0.742 g PDPM was obtained as a yellow powder with a yield of 61.8 %. ^{1}H NMR (400 MHz, $CDCl_3$): δ=7.63-6.84 (broad, -C6H4-, 4H), 2.26-1.64 (broad, -CCH3-, 3H) , 3.02-2.79 (broad, -OH, 1H). ^{13}C NMR (100 MHz, $CDCl_3$): δ=198.69, 146.96, 142.27, 131.21, 130.29, 129.21, 128.87, 127.64, 121.37, 78.58, 30.83, 28.79.

Synthesis of polytriphenylmethanol (PMPM). To one flame-dried 2-neck round bottom flask containing 0.288 g (12.0 mmol) of freshly peeled Mg scraps, was added 20 mL of dry THF. Then 1.990 g (10.0 mmol) 2-bromoacetophenone dissolved in 10 mL THF was added to the flask at room temperature through a syringe. After stirring for 5 min, 0.1 mL of 1, 2-dibromoethane was added to the flask as activator. After the reaction was refluxed for 24 h, the solution was cooled to room temperature, followed by quenching and hydrolysis with 20 mL saturated aqueous ammonium chloride.

After filtration and workup with dichloromethane/water, the organic solution was dried with anhydrous $MgSO_4$ and concentrated under reduced pressure. After the product was purified by precipitation into excessive petroleum ether, filtered and dried under vacuum, 0.813 g PMPM was obtained as a yellow powder with a yield of 67.7 %. ^{1}H NMR (400 MHz, $CDCl_3$): δ=7.74-6.73 (broad, -C6H5, 5H), 1.97-1.42 (broad, -CH2-, 2H) , 3.22-2.96 (broad, -OH, 1H) . ^{13}C NMR (100 MHz, $CDCl_3$): δ=198.78, 141.24, 136.59, 133.17, 128.14, 127.69, 126.93, 125.86, 59.61, 56.01, 41.22, 32.50, 29.97.

Synthesis of polyphenylmethanol (PXPM-1). To one flame-dried 2-neck round bottom flask containing 0.576 g (24.0 mmol) of freshly peeled Mg scraps, was added 20 mL of dry THF. Then 2.640 g (10.0 mmol) 1, 2-bis(bromomethyl)benzene and 2.242 g (10.0 mmol) dibenzoylmethane dissolved in 10 mL THF was added to the flask at room temperature through a syringe. After stirring for 5 min, 0.1 mL of 1, 2-dibromoethane was added to the flask as activator. After the reaction was refluxed for 24 h, the solution was cooled to room temperature, followed by quenching and hydrolysis with 20 mL saturated aqueous ammonium chloride. After filtration and workup with dichloromethane/water, the organic solution was dried with anhydrous $MgSO_4$ and concentrated under reduced pressure. After the product was purified by precipitation into excessive petroleum ether, filtered and dried under vacuum, 1.839 g PXPM-1 was obtained as a yellow solid with a yield of 55.7 %. ^{1}H NMR (400 MHz, $CDCl_3$): δ=8.04-7.70 (broad, -C6H5, 4H), 7.64-6.55 (broad, -C6H5, -C6H4-, 10H), 3.35-2.98 (broad, -COH, 2H), 2.93-2.51 (broad, -Ar-CH2-, 4H), 2.42-1.95 (broad,

-CH2-, 2H). ^{13}C NMR (100 MHz, $CDCl_3$): δ=185.43, 139.41, 135.58, 132.45, 130.24, 129.99, 128.69, 128.04, 127.18, 126.31, 61.99, 46.05, 34.67.

Synthesis of polyphenylmethanol (PXPM-2). To one flame-dried 2-neck round bottom flask containing 0.576 g (24.0 mmol) of freshly peeled Mg scraps, was added 20 mL of dry THF. Then 2.640 g (10.0 mmol) 1, 3-bis(bromomethyl)benzene and 2.242 g (10.0 mmol) dibenzoylmethane dissolved in 10 mL THF was added to the flask at room temperature through a syringe. After stirring for 5 min, 0.1 mL of 1, 2-dibromoethane was added to the flask as activator. After the reaction was refluxed for 24 h, the solution was cooled to room temperature, followed by quenching and hydrolysis with 20 mL saturated aqueous ammonium chloride. After filtration and workup with dichloromethane/water, the organic solution was dried with anhydrous $MgSO_4$ and concentrated under reduced pressure. After the product was purified by precipitation into excessive petroleum ether, filtered and dried under vacuum, 1.895 g PXPM-2 was obtained as a yellow solid with a yield of 57.4 %. ^1H NMR (400 MHz, $CDCl_3$): δ=8.12-7.69 (broad, -C6H5, 4H), 7.67-6.48 (broad, -C6H5, -C6H4-, 10H), 3.34-2.91 (broad, -COH, 2H), 2.40-2.12 (broad, -Ar-CH2-, 4H), 1.99-1.65 (broad, -CH2-, 2H). ^{13}C NMR (100 MHz, $CDCl_3$): δ=185.44, 137.10, 135.60, 133.66, 132.37, 130.24, 128.68, 128.03, 127.18, 126.70, 125.08, 50.20, 46.01, 41.78.

Synthesis of polyphenylmethanol (PXPM-3). To one flame-dried 2-neck round bottom flask containing 0.576 g (24.0 mmol) of freshly peeled Mg scraps, was added 20 mL of dry THF. Then 2.640 g (10.0 mmol) 1, 4-bis(bromomethyl)benzene and 2.242 g (10.0 mmol) dibenzoylmethane dissolved in 10 mL THF was added to the

flask at room temperature through a syringe. After stirring for 5 min, 0.1 mL of 1, 2-dibromoethane was added to the flask as activator. After the reaction was refluxed for 24 h, the solution was cooled to room temperature, followed by quenching and hydrolysis with 20 mL saturated aqueous ammonium chloride. After filtration and workup with dichloromethane/water, the organic solution was dried with anhydrous $MgSO_4$ and concentrated under reduced pressure. After the product was purified by precipitation into excessive petroleum ether, filtered and dried under vacuum, 1.882 g PXPM-3 was obtained as a yellow solid with a yield of 57.0 %. ^1H NMR (400 MHz, $CDCl_3$): δ=8.09-7.72 (broad, -C6H5, 4H), 7.68-6.50 (broad, -C6H5, -C6H4-, 10H), 3.36-2.94 (broad, -COH, 2H), 2.94-2.64 (broad, -CH2-Ar-CH2-, 4H), 2.38-2.12 (broad, -CH2-, 2H). ^{13}C NMR (100 MHz, $CDCl_3$): δ=185.73, 145.97, 137.03, 133.68, 133.13, 130.14, 128.67, 128.29, 127.98, 127.23, 125.09, 93.17, 49.88, 45.23, 40.88.

Synthesis of styryldiphenylmethanol (SDPM). To one flame-dried 2-neck round bottom flask containing 0.576 g (24 mmol) of freshly peeled Mg scraps, was added 10 mL of dry THF. Then 3.660 g (20 mmol) 4-bromostyrene dissolved in 10 mL THF was added to the flask at room temperature through a syringe. Then the reactions were refluxed for 3 h. After 3 h, 3.644 g (20 mmol) benzophenone dissolved in 10 mL THF was added dropwise to the flask at 0 °C through a syringe. Then the solution reacted continuely for 12 h at room temperature, followed by quenching and hydrolysis with 20 mL saturated aqueous ammonium chloride. After filtration and workup with dichloromethane/water, the organic solution was dried with anhydrous $MgSO_4$ and concentrated under reduced pressure. Then the crude product was subjected to column

chromatography on silica gel with petroleum ether/ethyl acetate mixture (3/1 v/v) as the eluent to afford 4.211 g SDPM with a yield of 73.6 %. ^{1}H NMR (400 MHz, $CDCl_3$): δ=7.32 (d, -C6H4-, 2H), 7.20-7.28 (m, -C6H5, 10H), 7.14 (d, -C6H4-, 2H), 6.68 (dd, -CHCH2, 1H), 5.71(d, -CHCH2, 1H), 5.21(d, -CHCH2, 1H), 2.75 (s, -C-OH, 1H). ^{13}C NMR (100 MHz, $CDCl_3$): δ=146.62, 144.22, 136.57, 136.39, 128.64, 128.14, 127.96, 127.33, 125.81, 114.13, 81.93.

Synthesis of polystyryldiphenylmethanol (PSDPM). The synthesis of PSDPM via RAFT polymerization was as follows: SDPM (1.145 g, 4 mmol), DMP (7.3 mg, 20 μmol), AIBN (0.3 mg, 30 μL of freshly prepared 10 mg/mL AIBN stock solution in THF, 2 μmol) and 1 mL THF were loaded into a Schlenk tube. Then the solution was degassed by three freeze-evacuate-thaw cycles. After the Schlenk tube was sealed, it was immersed in oil bath at 80 ^{o}C and kept stirring for 12 h. Polymerization was stopped by putting Schlenk tube in icy water. After the product was purified by precipitation into excessive petroleum ether, filtered and dried under vacuum, 0.72 g PSDPM was obtained as a white solid with a yield of 62.9 %. ^{1}H NMR (400 MHz, $CDCl_3$): δ=7.39-6.94 (broad, -C6H5, 10H), 6.94-6.67 (broad, -C6H4-, 2H), 6.67-6.16 (broad, -C6H4-, 2H), 3.36-2.87 (broad, -COH, 1H), 1.75-1.26 (broad, -CHCH2-, 3H). ^{13}C NMR (100 MHz, $CDCl_3$): δ=146.91, 144.34, 135.68, 128.62, 127.80, 127.29, 127.07, 125.79, 81.84, 40.20, 32.29.

Supplementary Figures

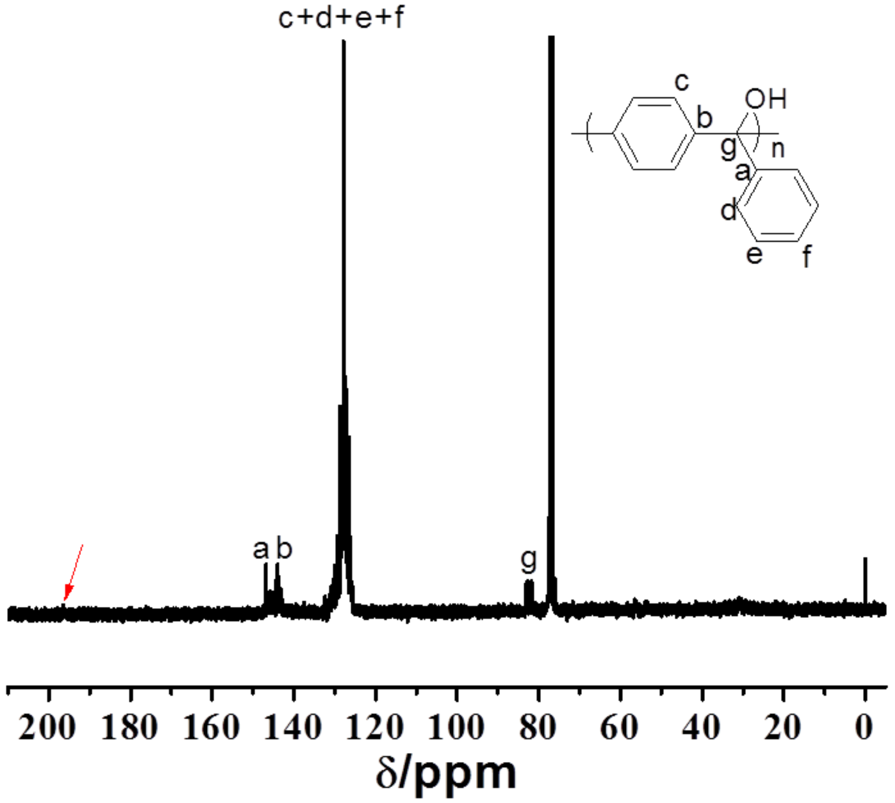

Supplementary Figure 1 ^{13}C NMR spectrum of PTPM-1 in $CDCl_3$ (the red arrow points to characteristic peak of carbonyl group).

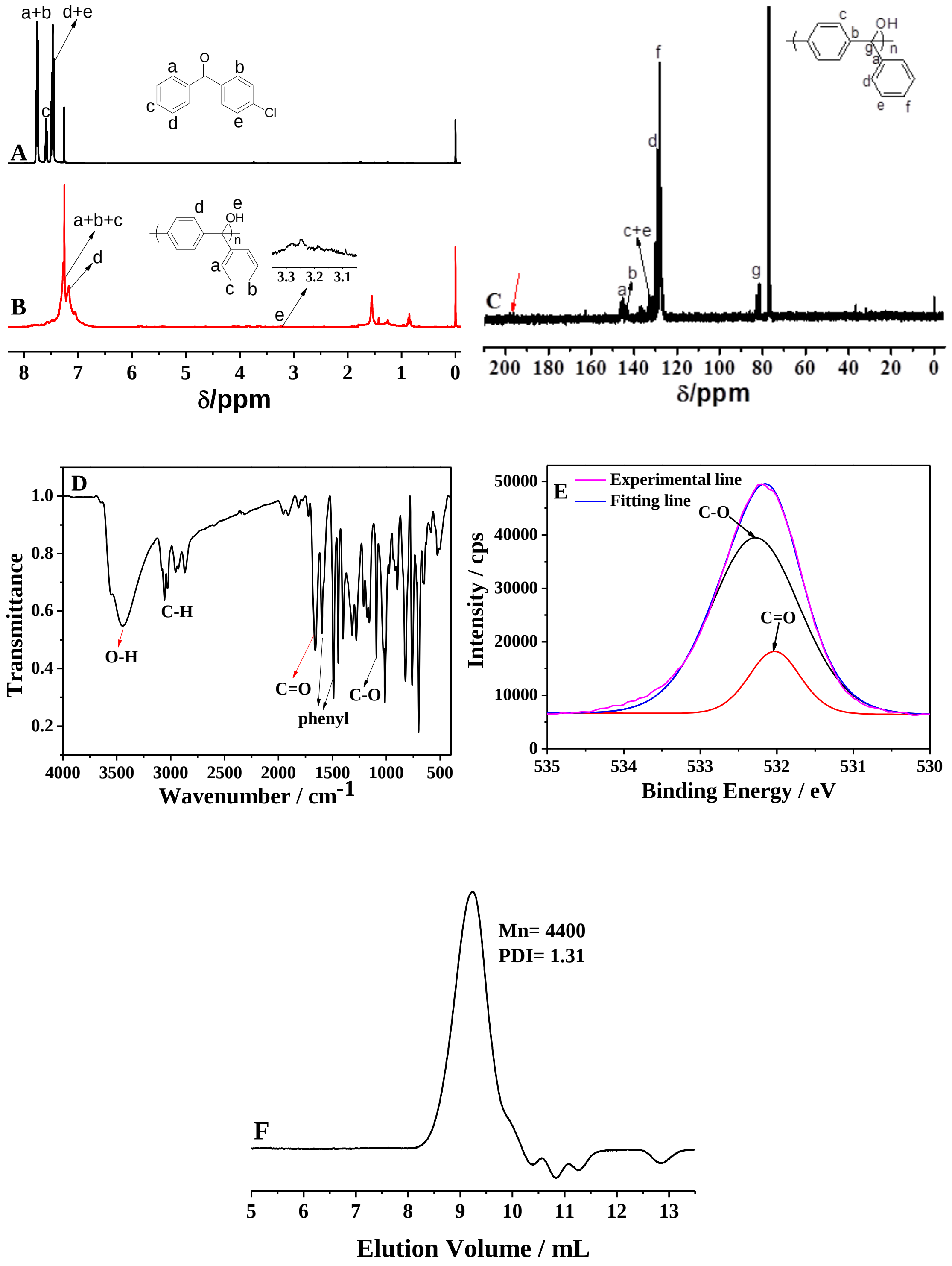

Supplementary Figure 2 ^{1}H NMR spectra of 4-chlorobenzophenone (A) and PTPM-1' (B) in $CDCl_3$, ^{13}C NMR spectrum of PTPM-1' (C) in $CDCl_3$ (the red arrow points to characteristic peak of carbonyl group), FT-IR spectrum of PTPM-1' (D), XPS O1s spectrum of PTPM-1' (E) and GPC curve of PTPM-1' (F).

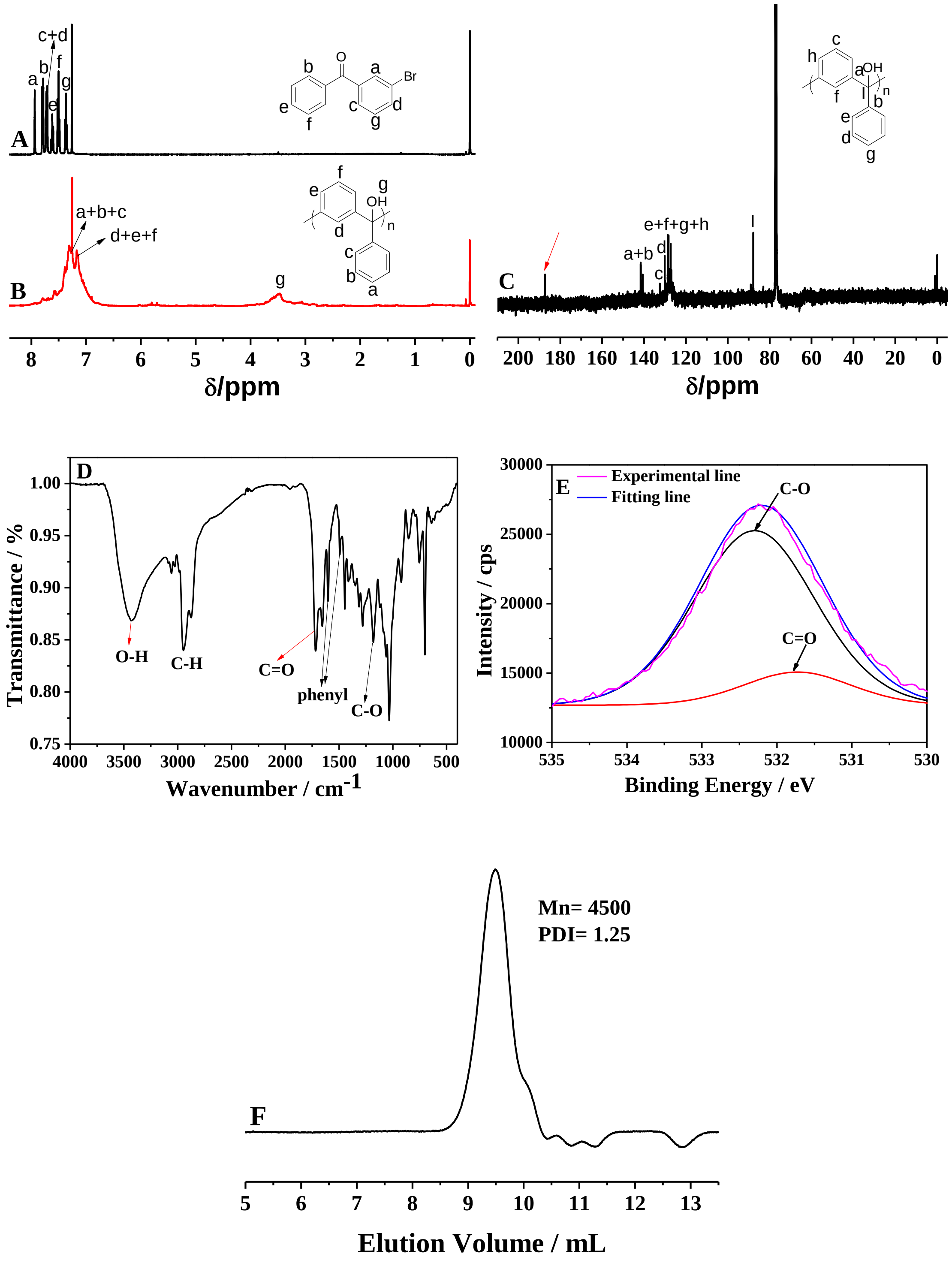

Supplementary Figure 3 ^{1}H NMR spectra of 3-bromobenzophenone (A) and PTPM-2 (B) in $CDCl_3$, ^{13}C NMR spectrum of PTPM-2 (C) in $CDCl_3$ (the red arrow points to characteristic peak of carbonyl group), FT-IR spectrum of PTPM-2 (D), XPS O1s spectrum of PTPM-2 (E) and GPC curve of PTPM-2 (F).

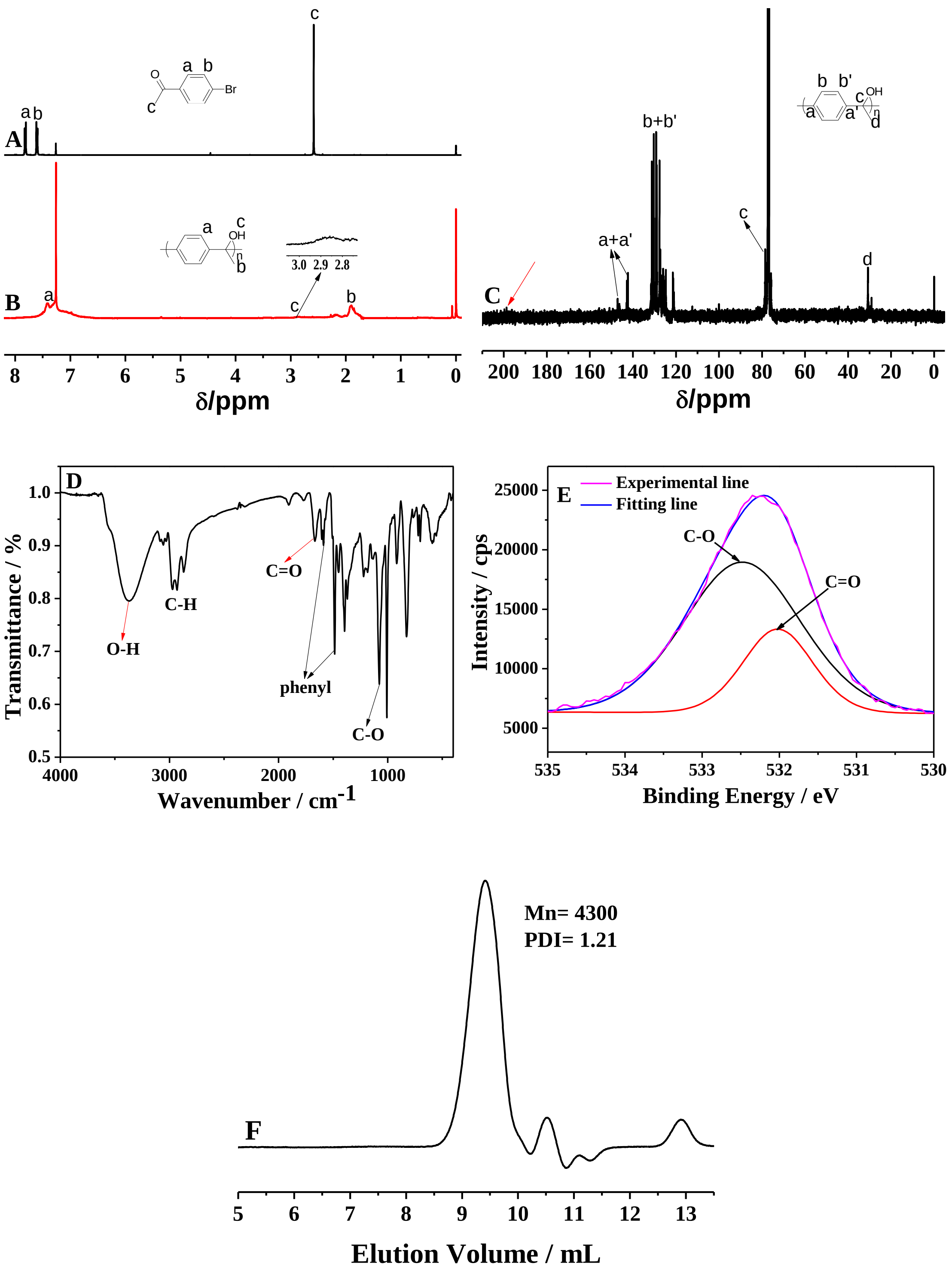

Supplementary Figure 4 ^{1}H NMR spectra of 4-bromoacetophenone (A) and PDPM (B) in $CDCl_3$, ^{13}C NMR spectrum of PDPM (C) in $CDCl_3$ (the red arrow points to characteristic peak of carbonyl group), FT-IR spectrum of PDPM (D), XPS O1s spectrum of PDPM (E) and GPC curve of PDPM (F).

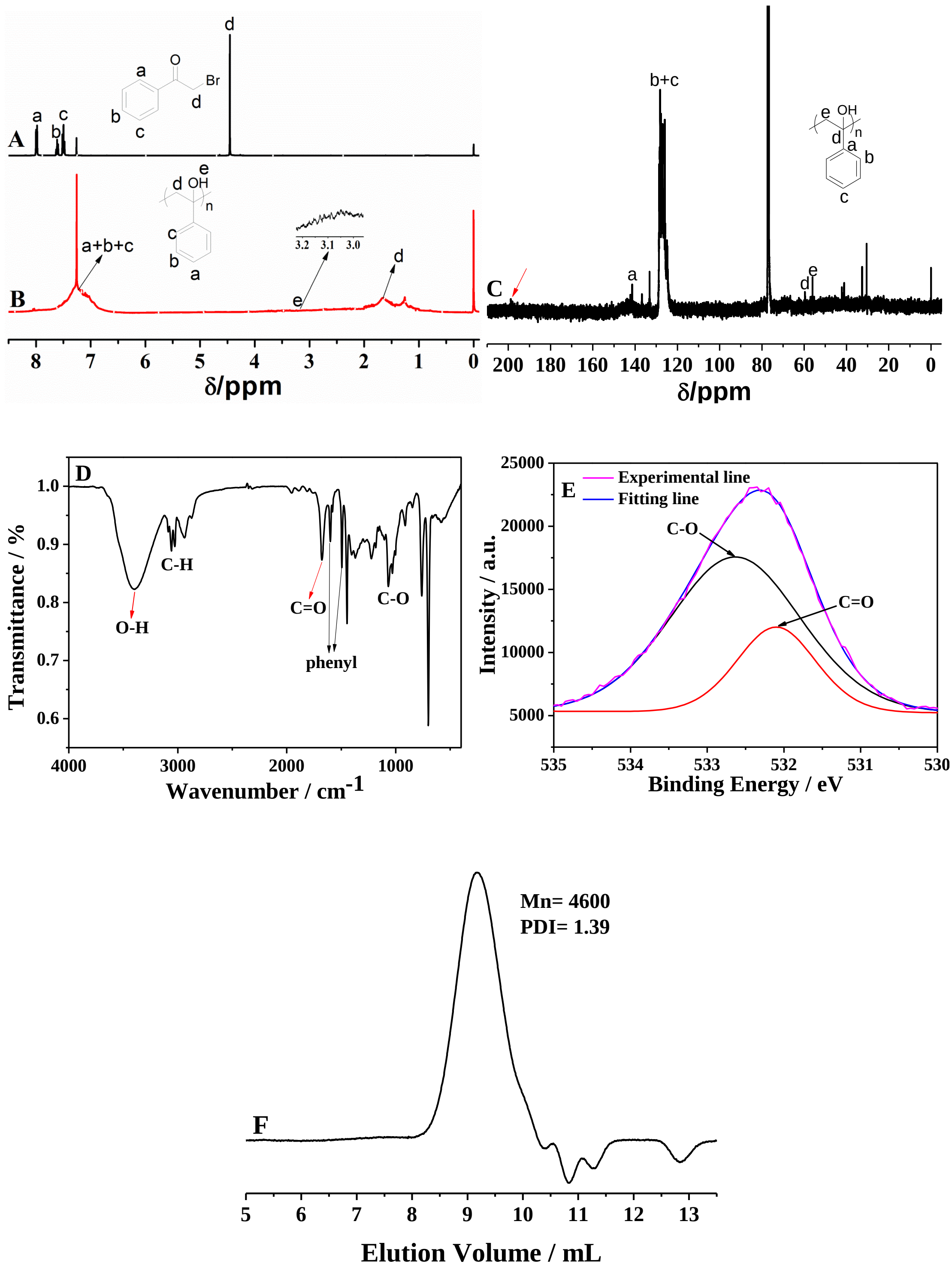

Supplementary Figure 5 ^{1}H NMR spectra of 2-bromoacetophenone (A) and PMPM (B) in $CDCl_3$, ^{13}C NMR spectrum of PMPM (C) in $CDCl_3$ (the red arrow points to characteristic peak of carbonyl group), FT-IR spectrum of PMPM (D), XPS O1s spectrum of PMPM (E) and GPC curve of PMPM (F).

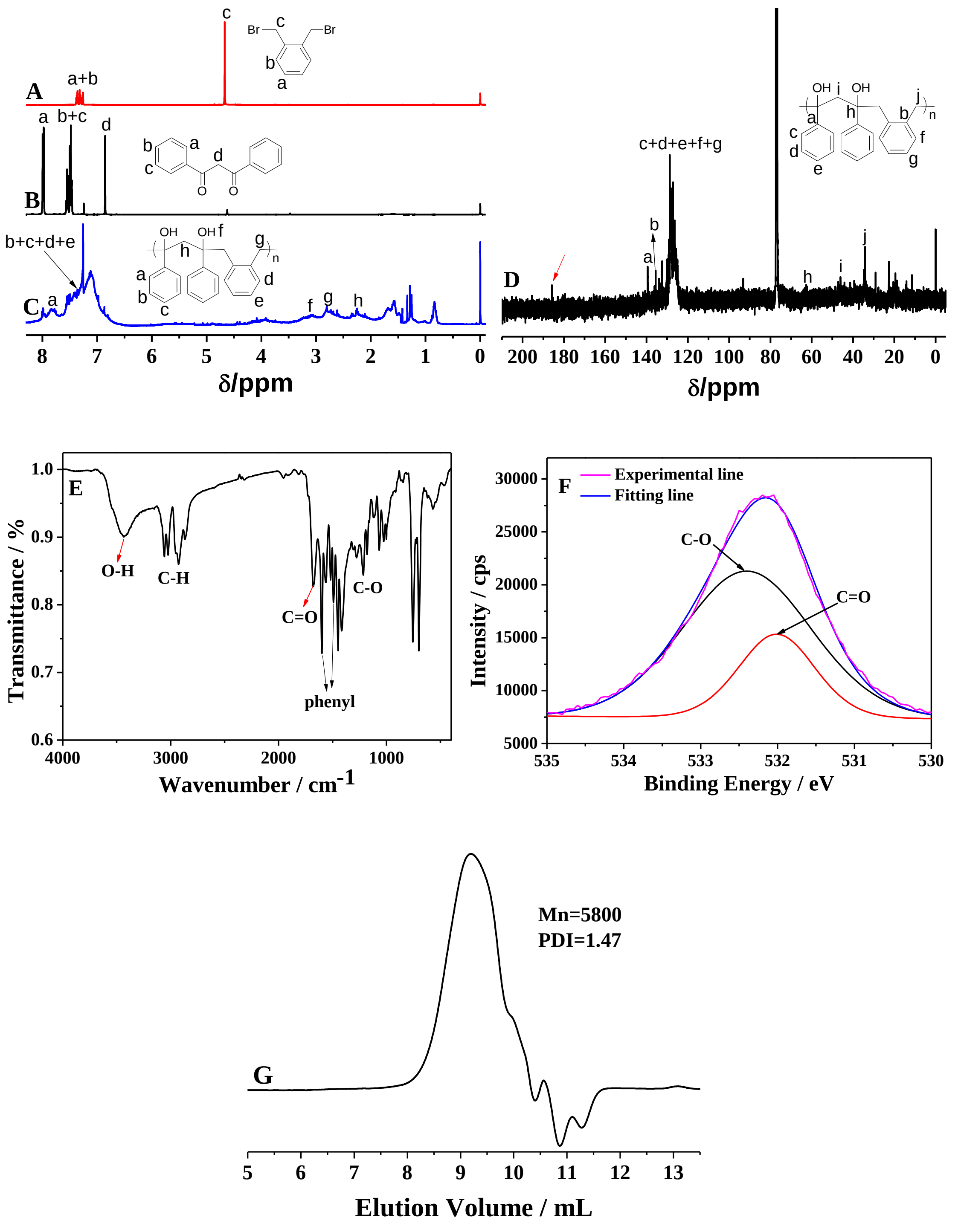

Supplementary Figure 6 ^{1}H NMR spectra of 1,2-bis(bromomethyl)benzene (A), dibenzoylmethane (B) and PXPM-1 (C) in $CDCl_3$, ^{13}C NMR spectrum of PXPM-1 (D) in $CDCl_3$ (the red arrow points to characteristic peak of carbonyl group), FT-IR spectrum of PXPM-1 (E), XPS O1s spectrum of PXPM-1 (F) and GPC curve of PXPM-1 (G).

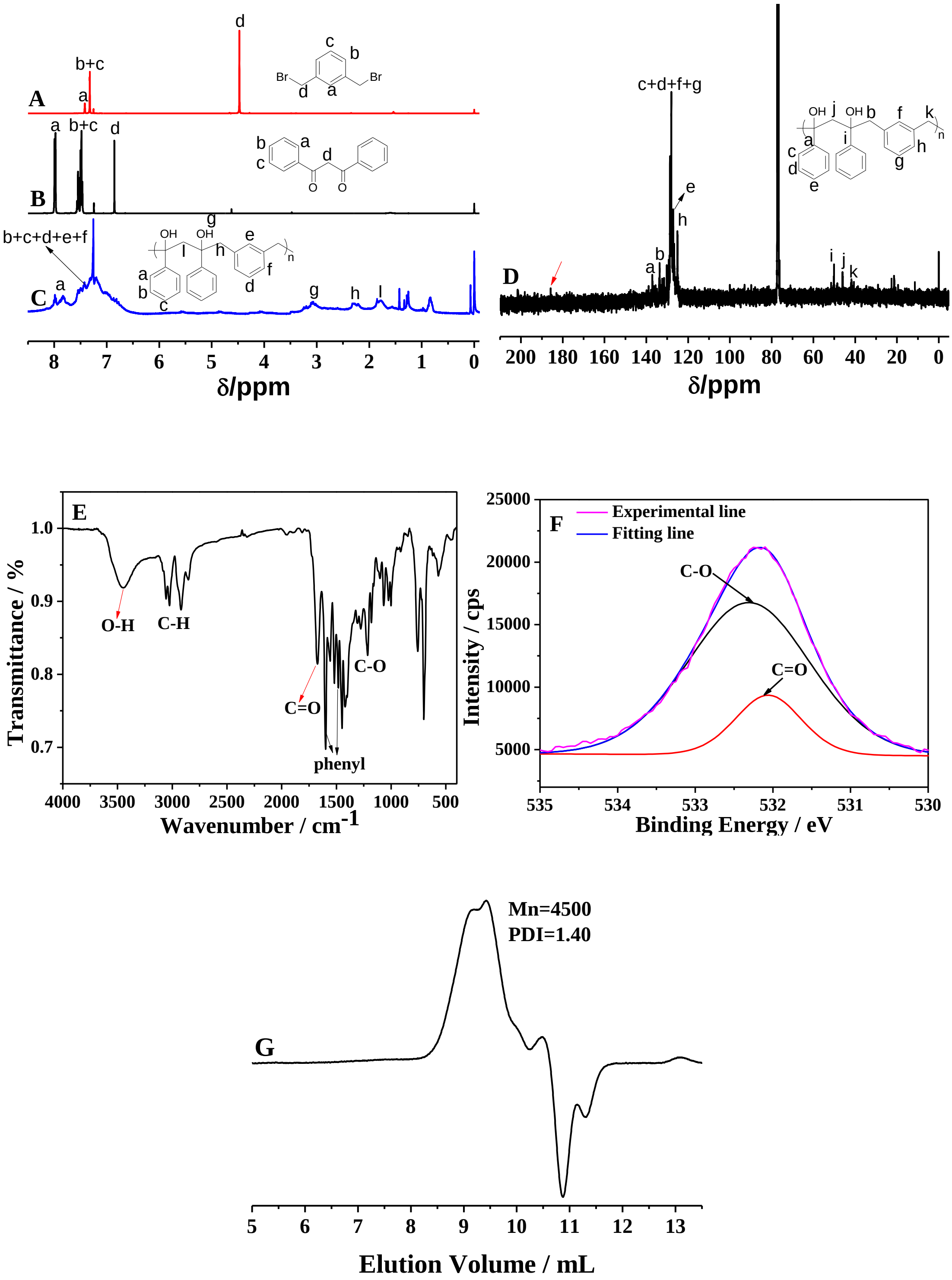

Supplementary Figure 7 ^{1}H NMR spectra of 1,3-bis(bromomethyl)benzene (A), dibenzoylmethane (B) and PXPM-2 (C) in $CDCl_3$, ^{13}C NMR spectrum of PXPM-2 (D) in $CDCl_3$ (the red arrow points to characteristic peak of carbonyl group), FT-IR spectrum of PXPM-2 (E), XPS O1s spectrum of PXPM-2 (F) and GPC curve of PXPM-2 (G).

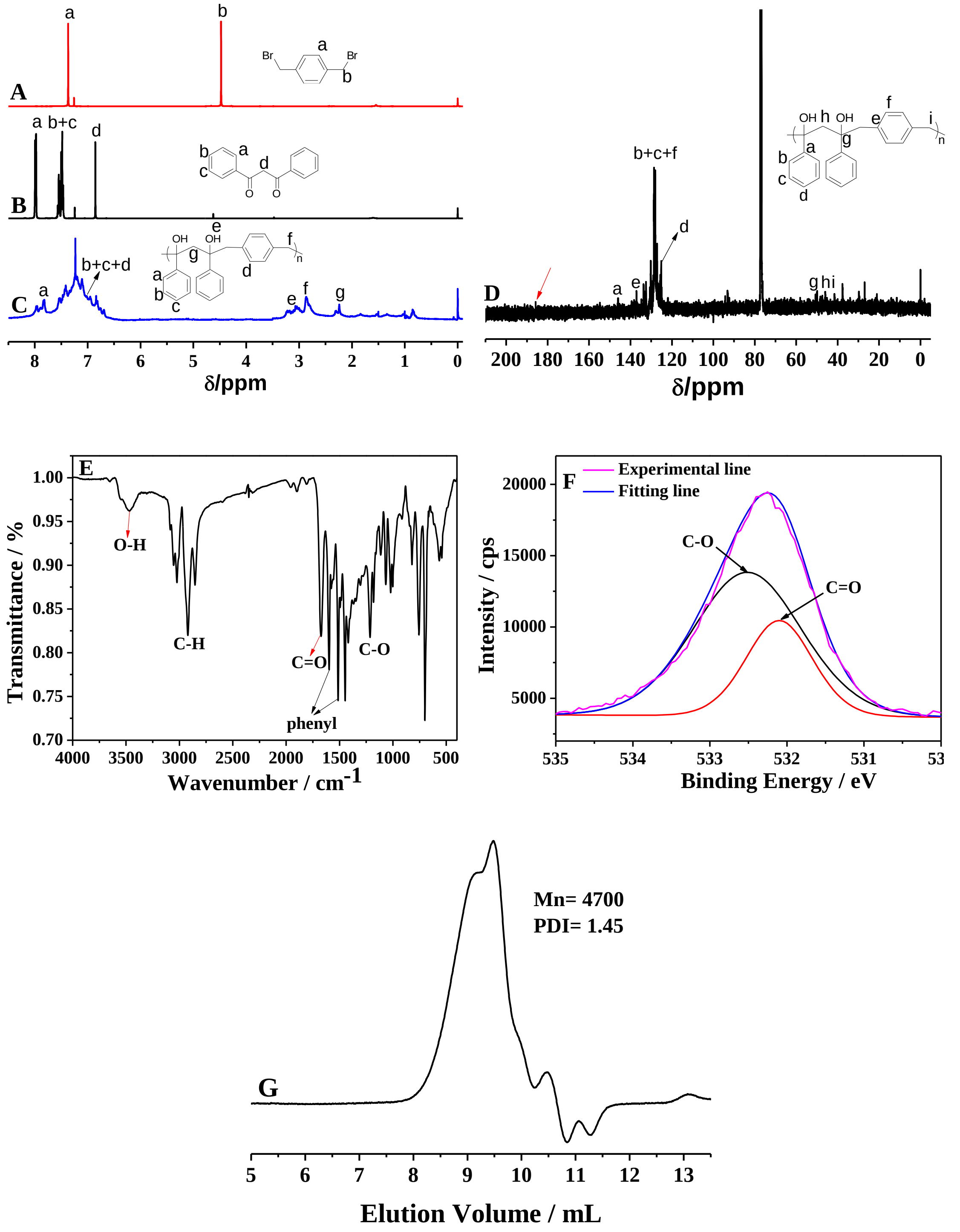

Supplementary Figure 8 ^{1}H NMR spectra of 1,4-bis(bromomethyl)benzene (A), dibenzoylmethane (B) and PXPM-3 (C) in $CDCl_3$, ^{13}C NMR spectrum of PXPM-3 (D) in $CDCl_3$ (the red arrow points to characteristic peak of carbonyl group), FT-IR spectrum of PXPM-3 (E), XPS O1s spectrum of PXPM-3 (F) and GPC curve of PXPM-3 (G).

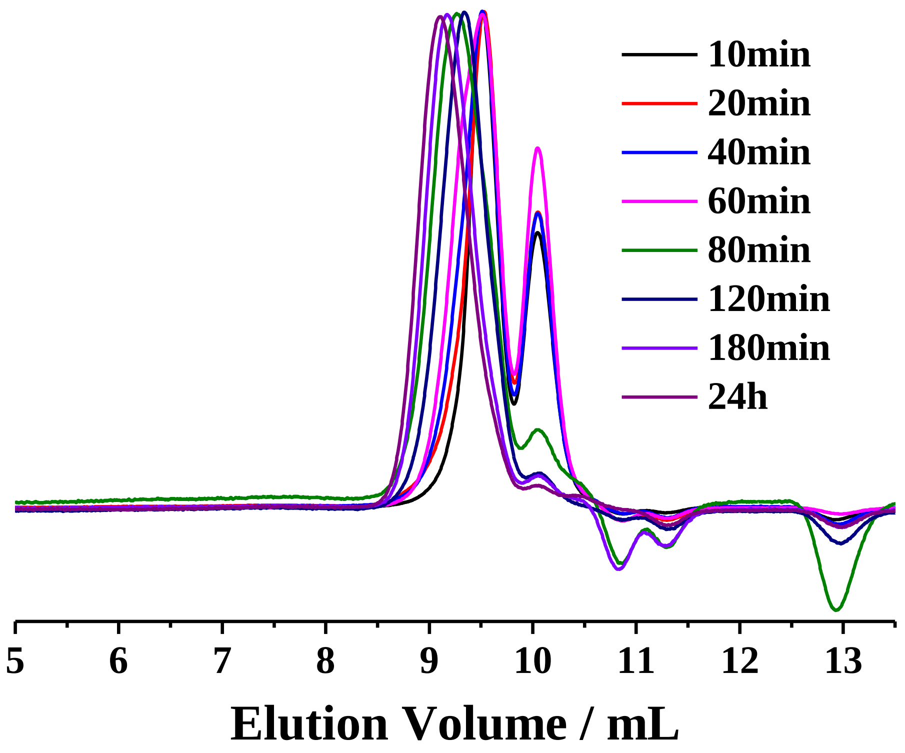

Supplementary Figure 9 GPC curves of PTPM-1 prepared by barbier polyaddition of 4-bromobenzophenone in THF at 80 °C.

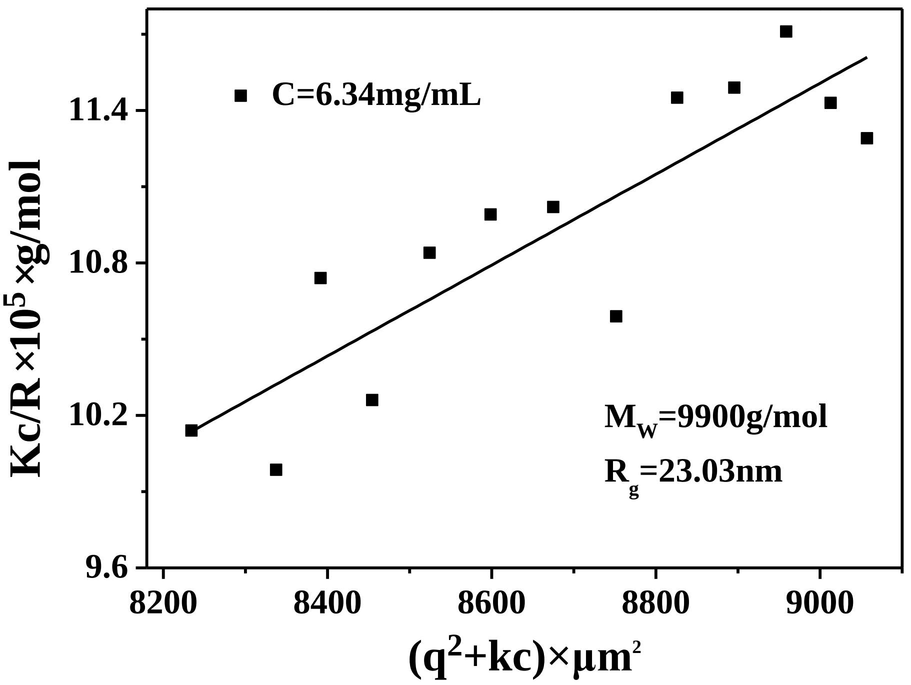

Supplementary Figure 10 Zimm plot of the PTPM-1 obtained by barbier polyaddition of 4-bromobenzophenone in THF at 80 °C.

Supplementary Table 1 The photophysical data of these model compounds

Entry	Substrate	Abbreviation	λ_{abs} (L)	λ_{ex} (L)	λ_{em} (L)	λ_{abs} (S)	λ_{ex} (S)	λ_{em} (S)	QY_L	QY_S	FL
1	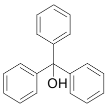	TPM	260	255	302	269	268	313	19.92	4.1	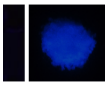
2	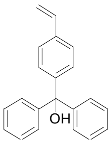	SDPM	255	264	306	296	326	458	31.91	17.44	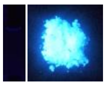
3	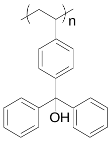	PSDPM	260	270	320	313	345	451	31.75	7.66	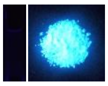
4	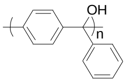	PTPM-1	259	274	318	308	358	465	22.25	11.98	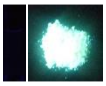
5	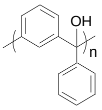	PTPM-2	258	270	314	—	—	—	6.17	—	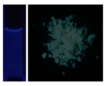
6	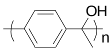	PDPM	258	—	—	309	—	—	—	—	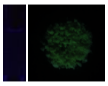
7	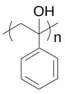	PMPM	259	—	—	317	—	—	—	—	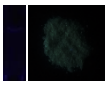
8	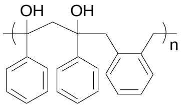	PXPM-1	345	—	—	359	—	—	—	—	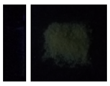
9	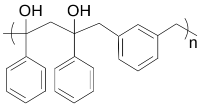	PXPM-2	348	—	—	329	—	—	—	—	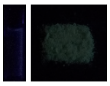
10	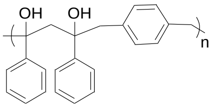	PXPM-3	338	—	—	338	—	—	—	—	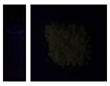

λ_{abs}(L), λ_{ex}(L) and λ_{em}(L) refer to optimal absorption wavelength, optimal excitation wavelength and optimal emission wavelength of the liquid sample respectively. λ_{abs}(S), λ_{ex}(S) and λ_{em}(S) refers to optimal absorption wavelength, optimal excitation wavelength and optimal emission wavelength of the solid sample respectively. QY_L and QY_S refer to fluorescence quantum yield of the liquid and solid sample respectively. FL refers to fluorescence emission properties (under irradiation with UV lamp @365 nm) of the liquid and solid sample. THF was chosen as the solvent used in the measurement of results in Supplementary Table 1.

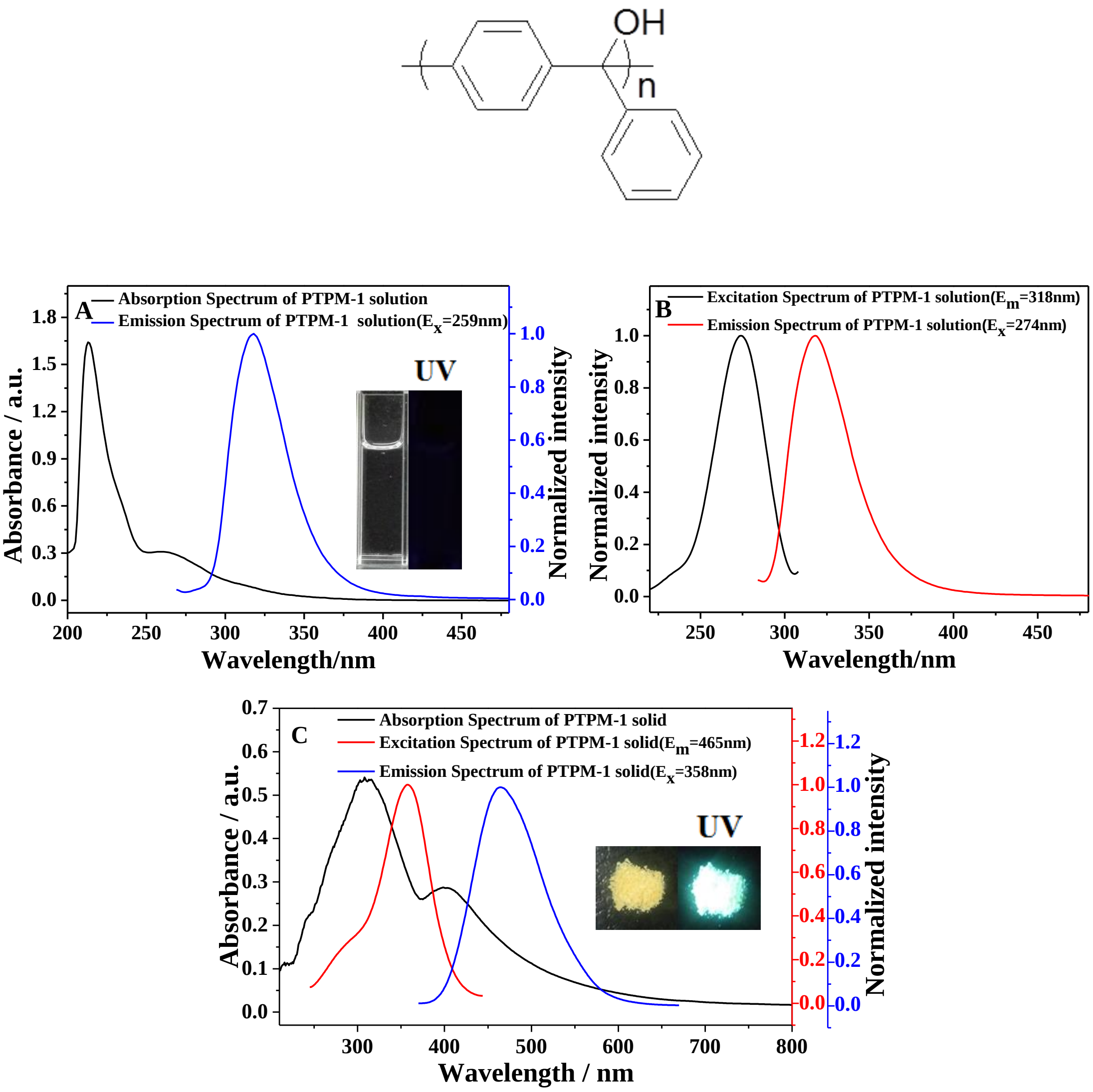

Supplementary Figure 11 The photophysical properties of PTPM-1. A) absorption spectrum, emission spectrum (excited @ 259 nm) of solution and digital photos (under sunlight and irradiation with UV lamp @ 365 nm) of solution, B) excitation spectrum and emission spectrum (excited @ 274nm) of solution and C) absorption spectrum, excitation spectrum and emission spectrum (excited @ 358nm) of solid and digital photos (under sunlight and irradiation with UV lamp @ 365 nm) of solid. THF was chosen as the solvent used in the measurement.

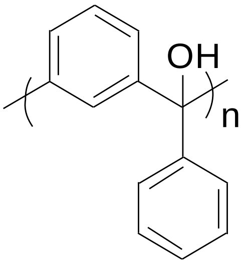

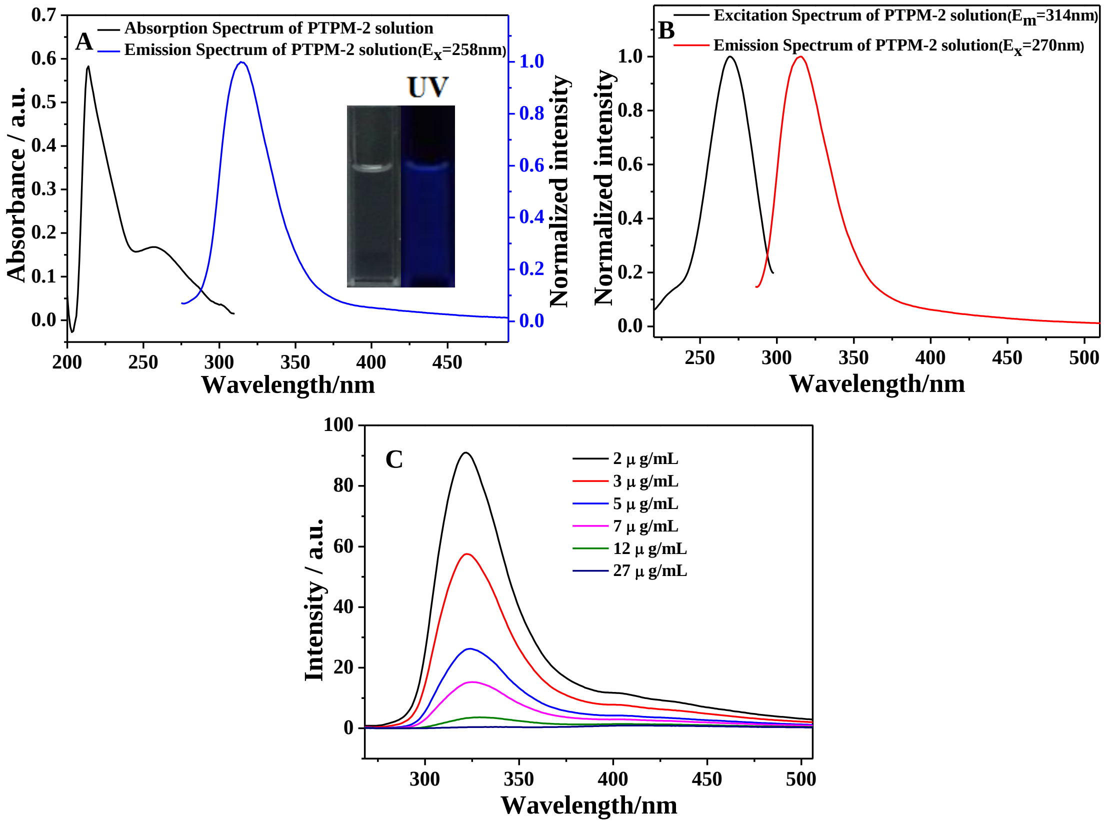

Supplementary Figure 12 The photophysical properties of PTPM-2. A) absorption spectrum, emission spectrum (excited @ 258 nm) of solution and digital photos (under sunlight and irradiation with UV lamp @ 365 nm) of solution, B) excitation spectrum and emission spectrum (excited @ 270nm) of solution and C) the solution fluorescence quenching curve(excited @ 258 nm). THF was chosen as the solvent used in the measurement.

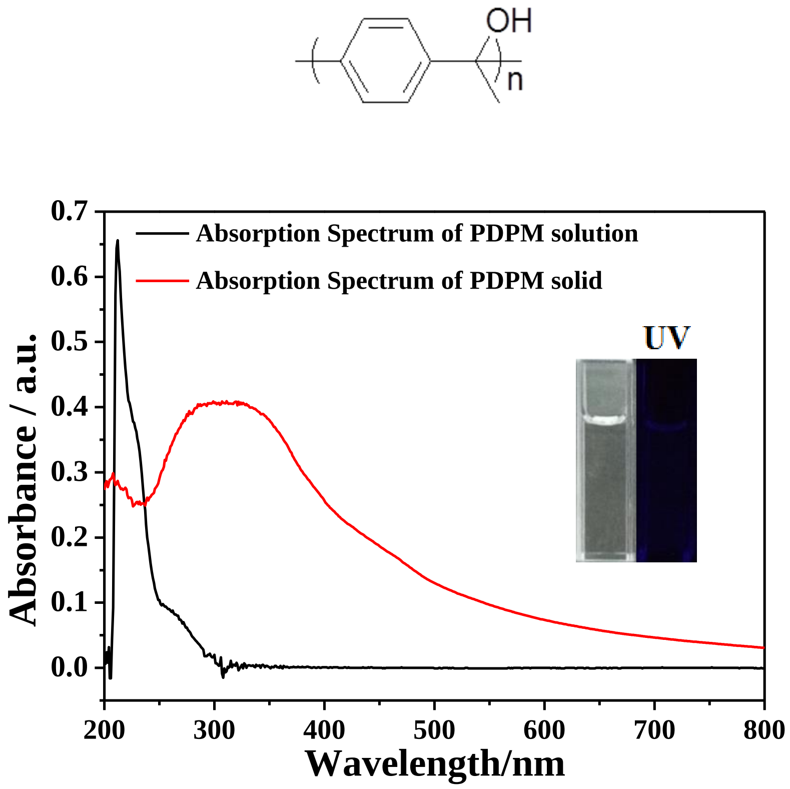

Supplementary Figure 13 The absorption spectra of PDPM solution and PDPM solid and digital photos (under sunlight and irradiation with UV lamp @ 365 nm) of solution. THF was chosen as the solvent used in the measurement.

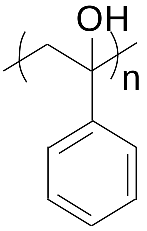

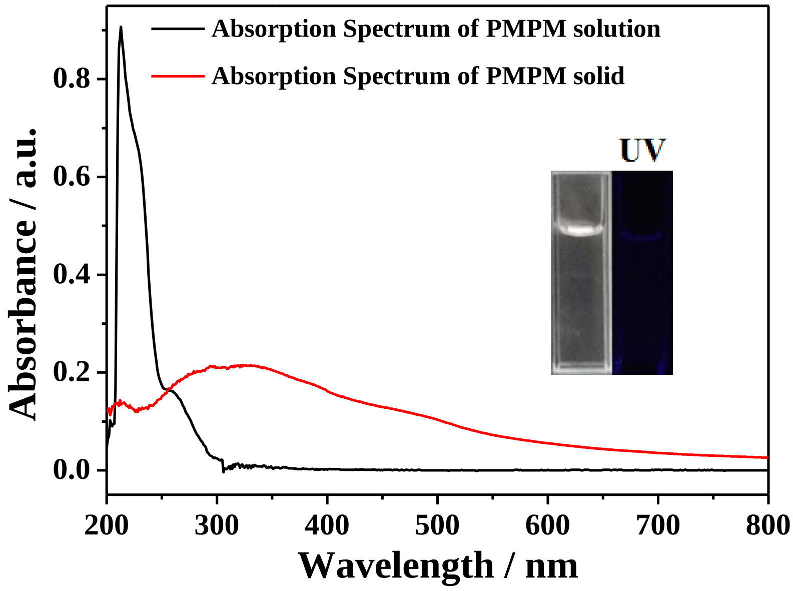

Supplementary Figure 14 The absorption spectra of PMPM solution and PMPM solid and digital photos (under sunlight and irradiation with UV lamp @ 365 nm) of solution. THF was chosen as the solvent used in the measurement.

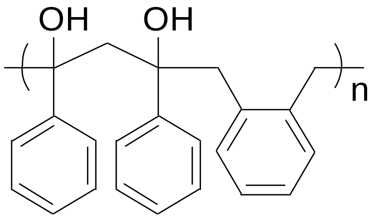

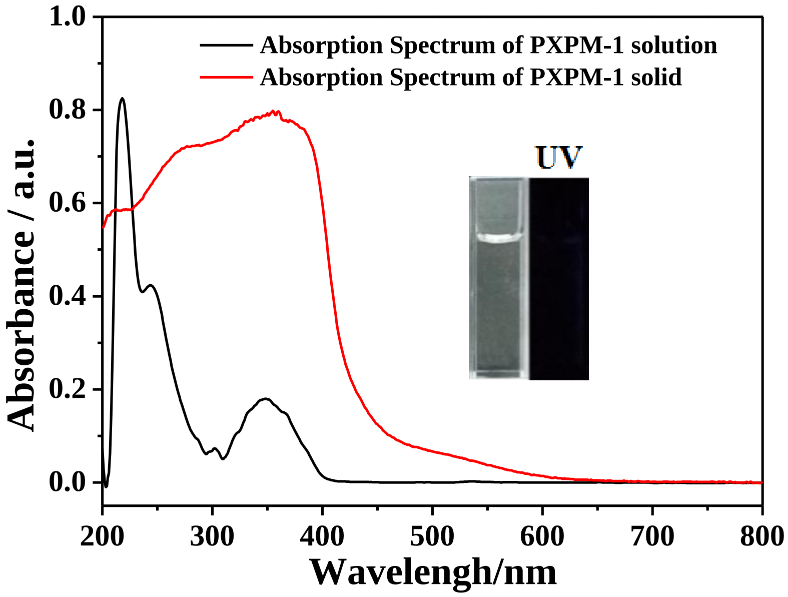

Supplementary Figure 15 The absorption spectrum of PXPM-1 solution and PXPM-1 solid and digital photos (under sunlight and irradiation with UV lamp @ 365 nm) of solution. THF was chosen as the solvent used in the measurement.

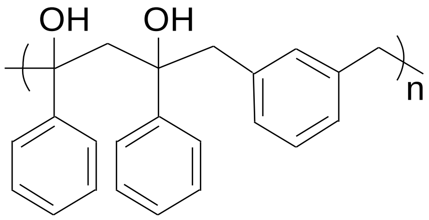

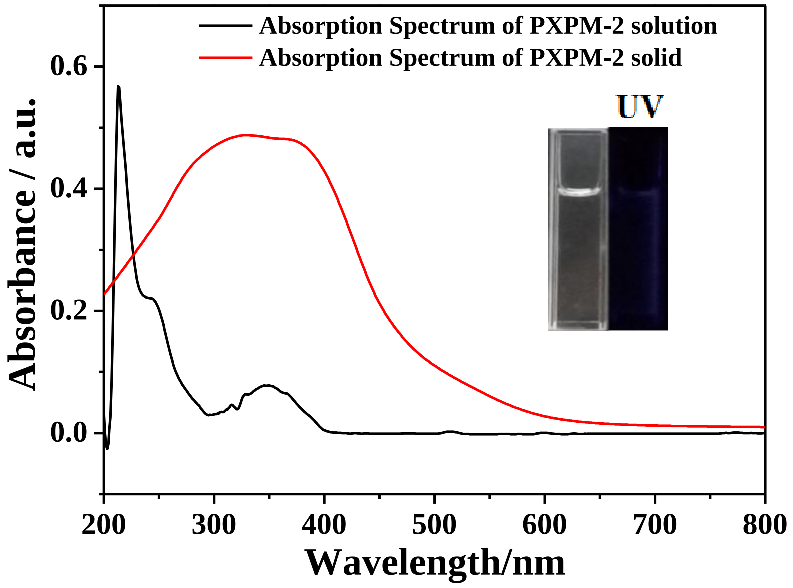

Supplementary Figure 16 The absorption spectrum of PXPM-2 solution and PXPM-2 solid and digital photos (under sunlight and irradiation with UV lamp @ 365 nm) of solution. THF was chosen as the solvent used in the measurement.

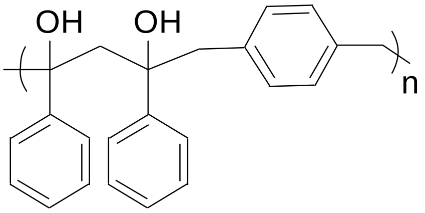

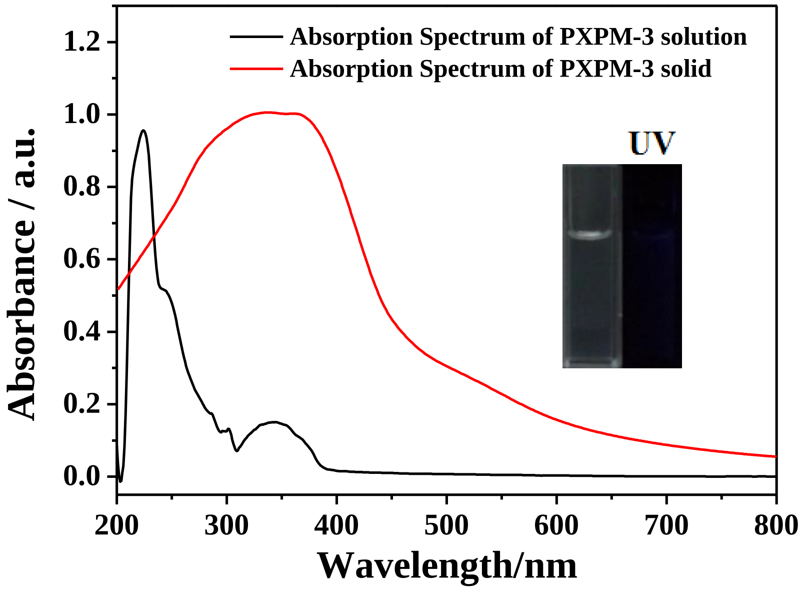

Supplementary Figure 17 The absorption spectrum of PXPM-3 solution and PXPM-3 solid and digital photos (under sunlight and irradiation with UV lamp @ 365 nm) of solution. THF was chosen as the solvent used in the measurement.

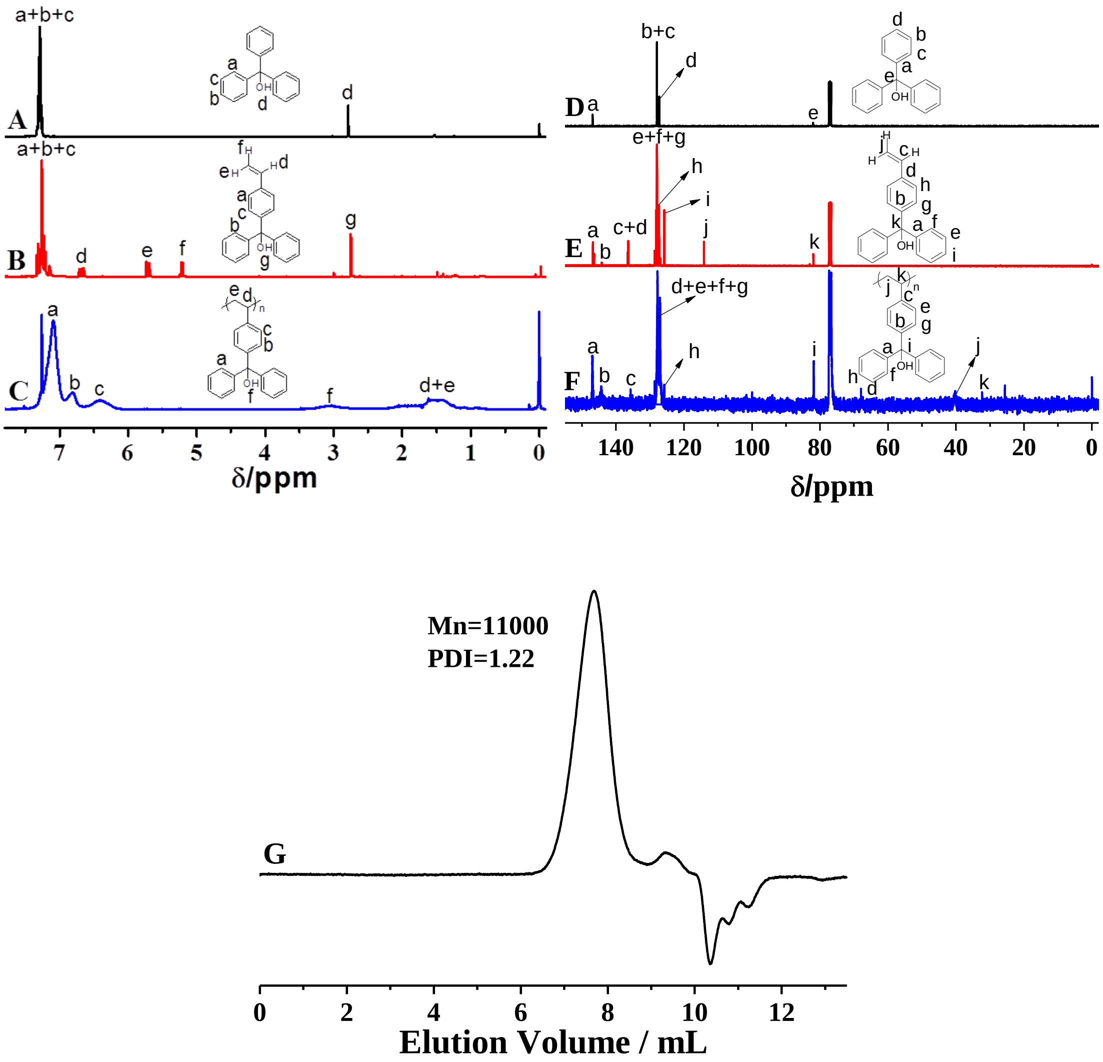

Supplementary Figure 18 ^{1}H NMR spectra of TPM (A) , SDPM (B) and PSDPM (C) in $CDCl_3$, ^{13}C NMR spectra of TPM (D) , SDPM (E) and PSDPM (F) in $CDCl_3$ and GPC curve of PSDPM (G).

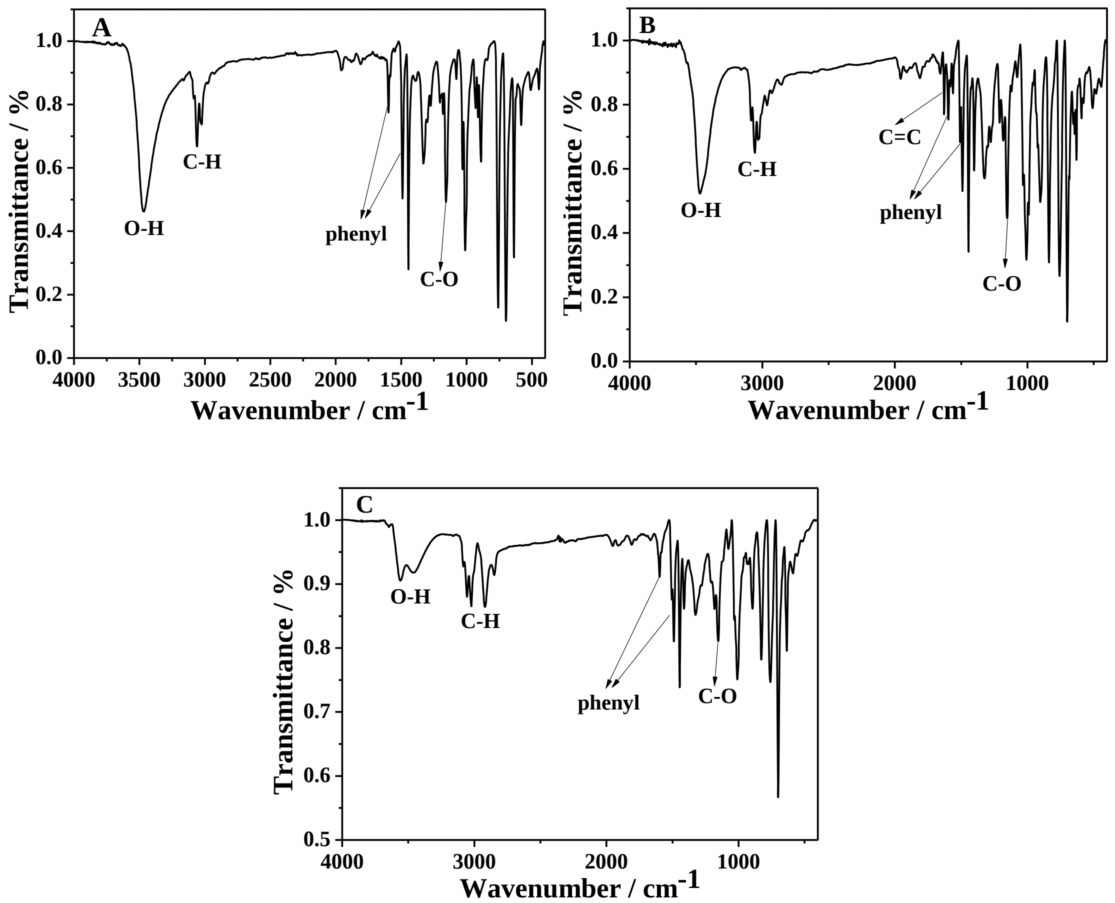

Supplementary Figure 19 FT-IR spectra of TPM (A), SDPM (B) and PSDPM (C) solids.

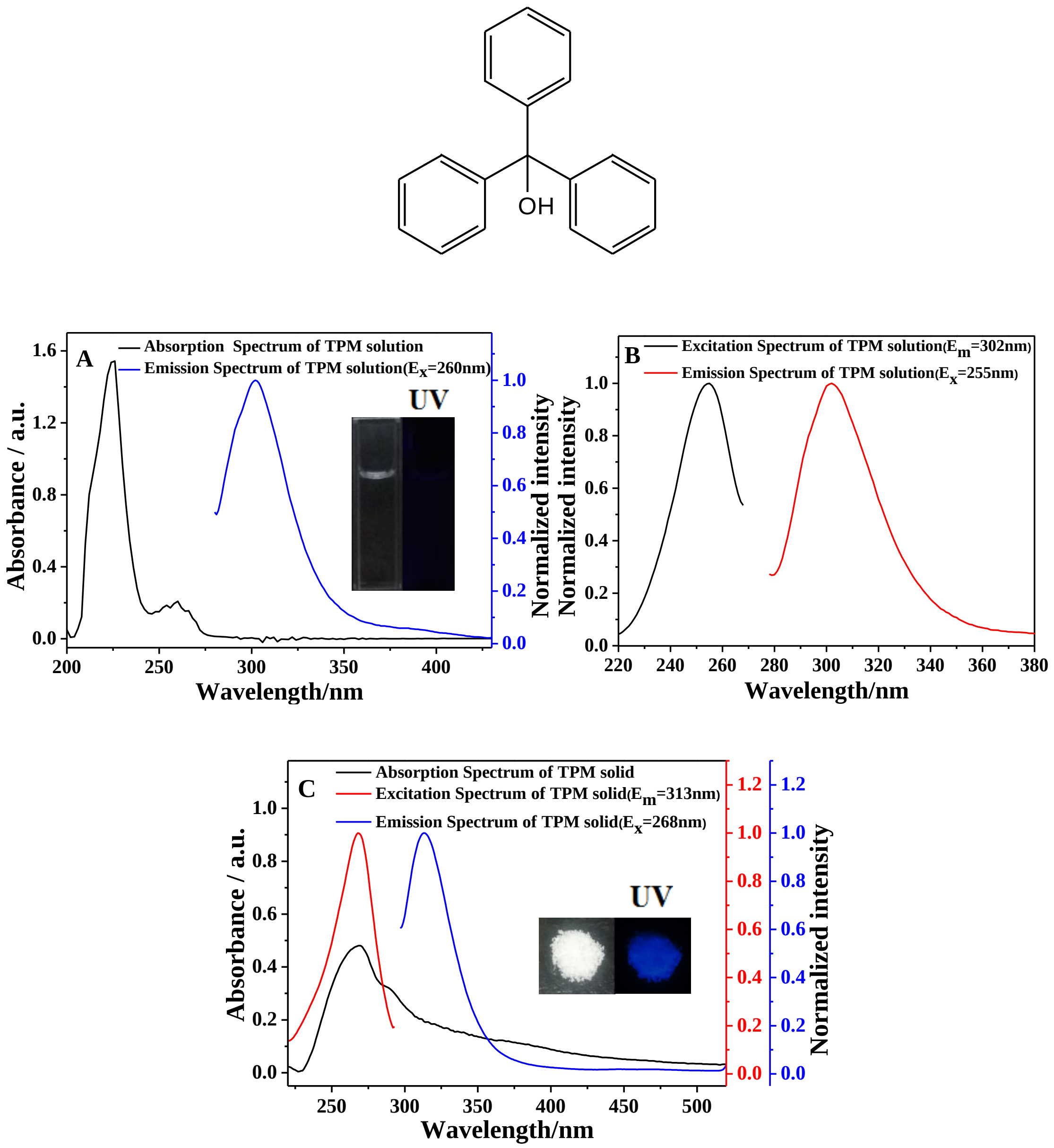

Supplementary Figure 20 The photophysical properties of TPM. A) absorption spectrum, emission spectrum (excited @ 260 nm) of solution and digital photos (under sunlight and irradiation with UV lamp @ 365 nm) of solution, B) excitation spectrum and emission spectrum (excited @ 255 nm) of solution and C) absorption spectrum, excitation spectrum and emission spectrum (excited @ 268 nm) of solid and digital photos (under sunlight and irradiation with UV lamp @ 365 nm) of solid. THF was chosen as the solvent used in the measurement.

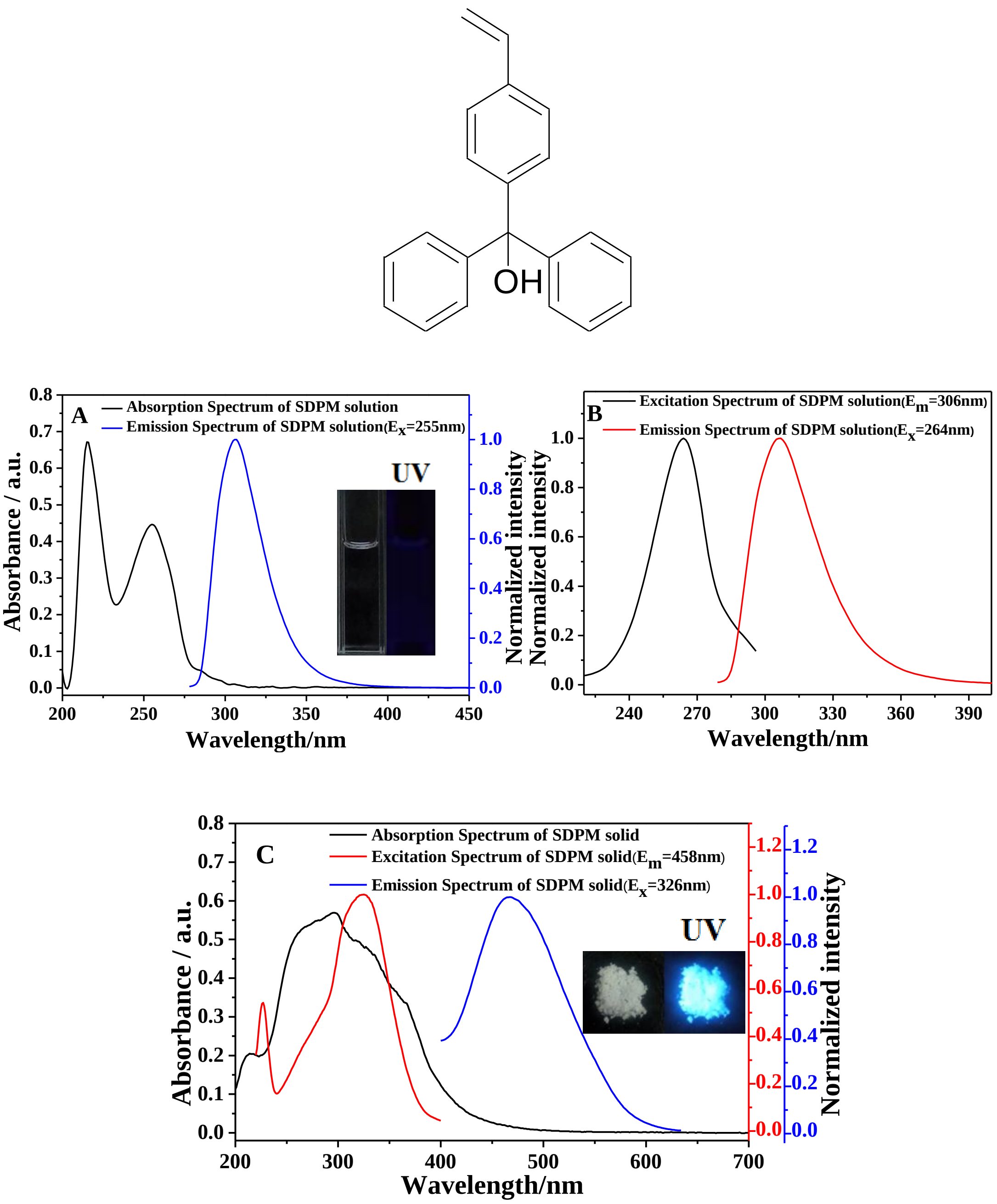

Supplementary Figure 21 The photophysical properties of SDPM. A) absorption spectrum, emission spectrum (excited @ 255 nm) of solution and digital photos (under sunlight and irradiation with UV lamp @ 365 nm) of solution, B) excitation spectrum and emission spectrum (excited @ 264nm) of solution and C) absorption spectrum, excitation spectrum and emission spectrum (excited @ 326nm) of solid and digital photos (under sunlight and irradiation with UV lamp @ 365 nm) of solid. THF was chosen as the solvent used in the measurement.

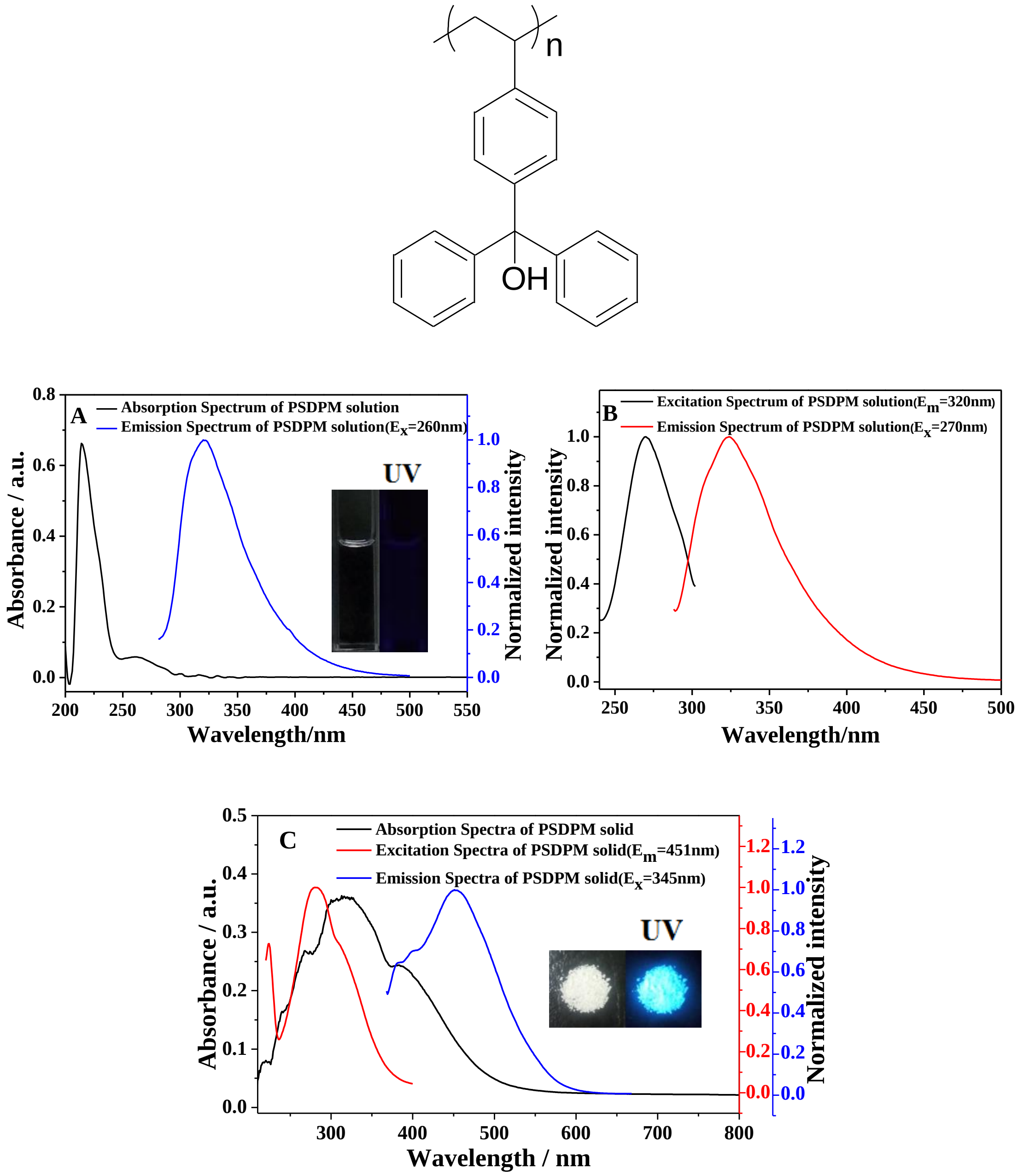

Supplementary Figure 22 The photophysical properties of PSDPM. A) absorption spectrum, emission spectrum (excited @ 260 nm) of solution and digital photos (under sunlight and irradiation with UV lamp @ 365 nm) of solution, B) excitation spectrum and emission spectrum (excited @ 270nm) of solution and C) absorption spectrum, excitation spectrum and emission spectrum (excited @ 345nm) of solid and digital photos (under sunlight and irradiation with UV lamp @ 365 nm) of solid. THF was chosen as the solvent used in the measurement.

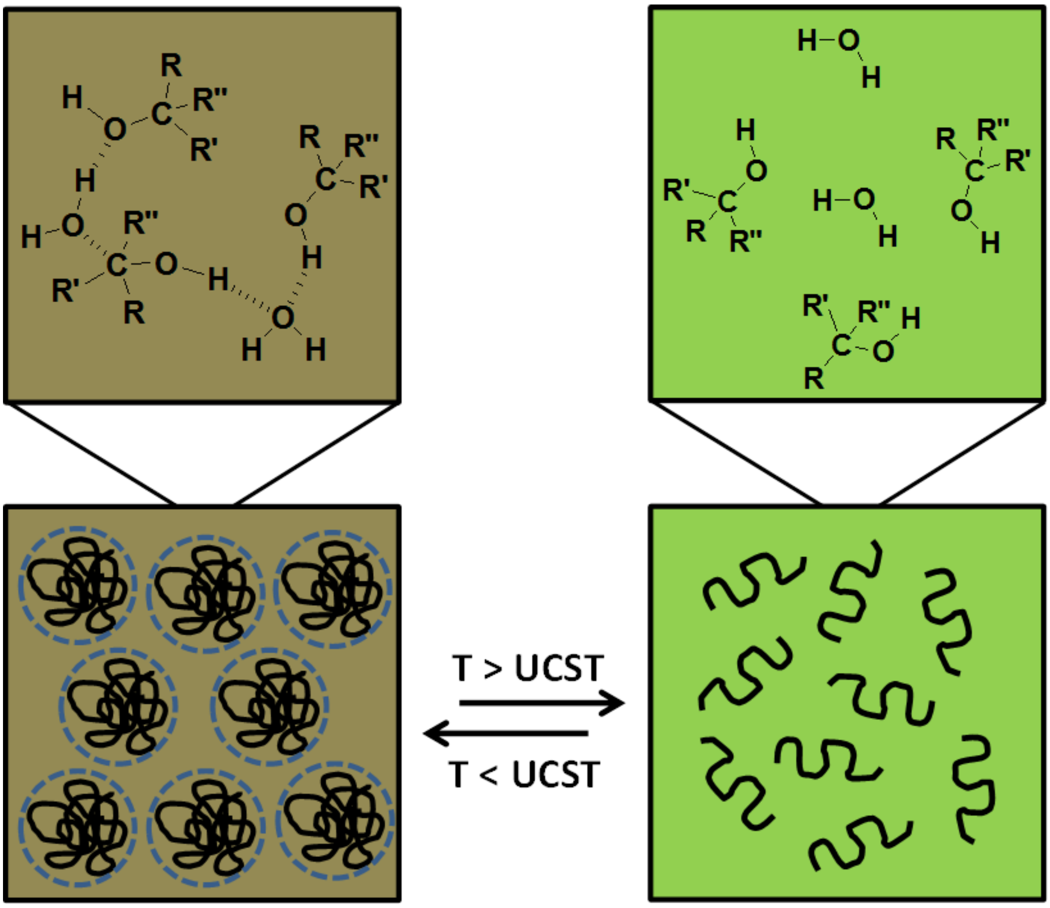

Supplementary Figure 23 Illustration of the proposed mechanism of the thermo-responsive behavior of PTPM-1
